# Circular RNA PRKCI (hsa_circ_0067934): a potential target in the pathogenesis of human malignancies

**DOI:** 10.3389/fonc.2024.1365032

**Published:** 2024-04-29

**Authors:** Shipei Qiu, Kefan Zhang, Siyu Chen, Shuting Yin

**Affiliations:** ^1^Department of General Surgery, Southeast University Affiliated Zhongda Hospital, Nanjing, China; ^2^Department of Cardiothoracic Center, The Second Affiliated Hospital of Nanjing Medical University, Nanjing, China; ^3^Department of Intensive Care Unit, Nanjing Drum Tower Hospital, The Affiliated Hospital of Nanjing University Medical School, Nanjing, China; ^4^Department of General Surgery, The Second Affiliated Hospital of Nanjing Medical University, Nanjing, China

**Keywords:** CircPRKCI, malignant tumor, molecular target, overall survival, invasion, migration and proliferation

## Abstract

Circular RNAs (circRNAs) are a new type of endogenous non-coding RNA formed by a covalent closed loop. CircRNAs are characterized by specificity, universality, conservation, and stability. They are abundant in eukaryotic cells and have biological regulatory roles at various transcriptional and post-transcriptional levels. The upregulation of circPRKCI has been observed in a variety of tumors and is directly related to the clinicopathological characteristics of tumors and prognosis. More importantly, circPRKCI can participate in the tumorigenesis, progression, recurrence, and metastasis of various tumors through many functional mechanisms, including the activation of signaling pathways, such as the phosphatidylinositol-3-kinase (PI3K)/AKT pathway, and sponging of many microRNAs (miRNAs). This review summarizes the progress achieved in understanding the biological functions of circRNA PRKCI in various tumors. The goal is to inform the discovery of more functional mechanisms and new anticancer molecular targets.

## 1 Background

### 1.1 Introduction

**Figure 1:**
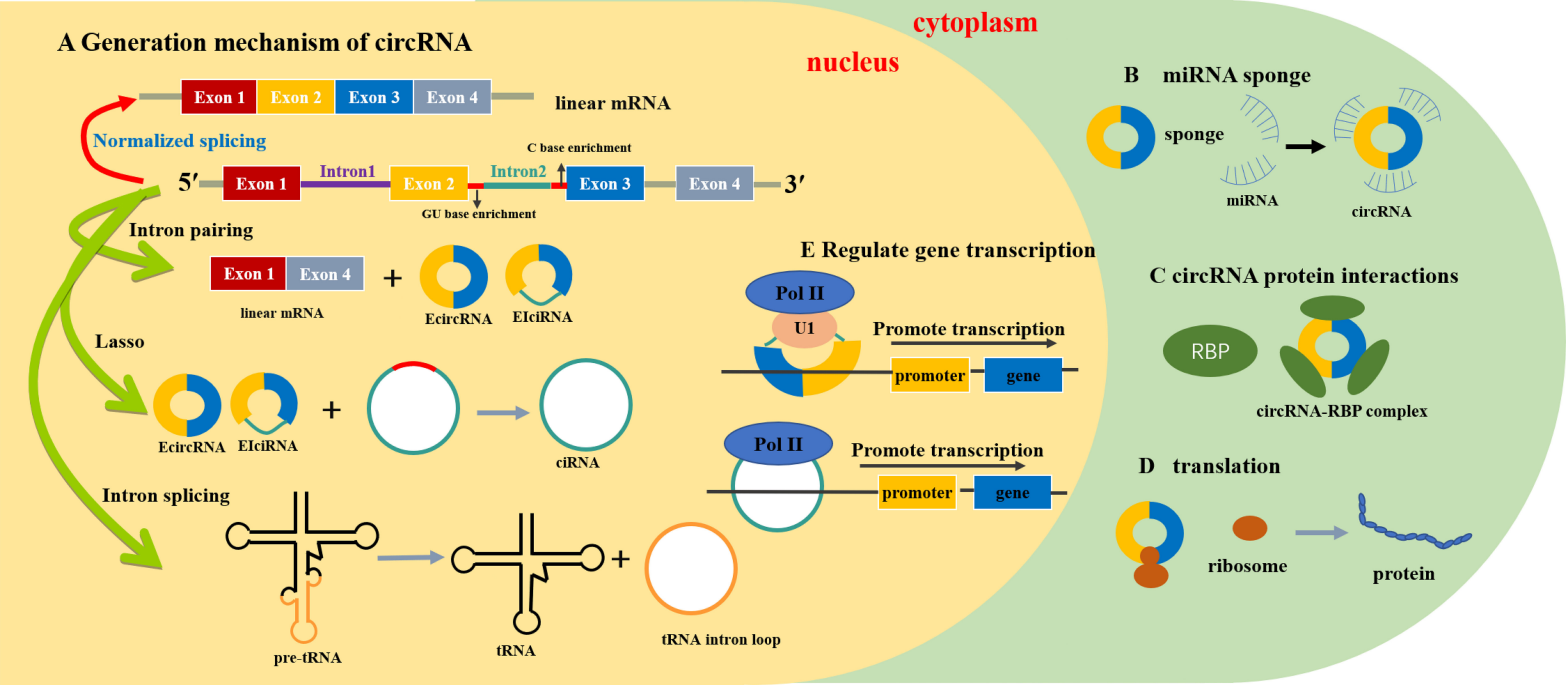
Generation mechanism and functional characteristics of circRNA (23). **(A)** Generation mechanism of circRNA. **(B)** circRNA act as miRNA sponge. **(C)** circRNA protein interaction. **(D)** circRNA promotes protein translation. **(E)** circRNA regulates gene transcription. mRNA, messenger RNA; tRNA, transport RNA; miRNA, microRNA; EcircRNA, exon circRNA; EIciRNA, exon–intron circRNA; ciRNA, intron circRNA; Pol II, RNA polymerase II; U1, anti-U1 small ribonucleoprotein antibody; RBP, RNA-binding protein.

Circular RNAs (circRNAs) comprise a large class of non-coding RNAs that are mainly formed by end-to-end connection of downstream 5′ and upstream 3′ splice sites produced by back-splicing or lasso introns of introns or exons (1). CircRNA was first observed in the cytoplasm of eukaryotic cells using electron microscopy in 1979 and was subsequently demonstrated to be pathogenic and infectious in higher plants (2). CircRNAs were first reported in 1986 and are considered the product of splicing errors, which have not been intensively studied (3). With the development of RNA sequencing (RNA-seq) technologies, circRNA has been widely observed in eukaryotic cells and plays an important role in regulating gene expression (4). CircPRKCI is widely expressed in tissues and plasma, such as the liver, lungs, thyroid, bladder, brain, uterus, stomach, esophagus, and other organs, and has a more stable and conserved sequence than other non-coding RNA. **Figure 1** summarizes the production mechanism and functional characteristics of circRNA.

CircPRKCI, also known as hsa_circ_0067934, is a circRNA that is encoded by the 3q26.2 amplicon of PRKCI. In recent years, there has been an increasing number of studies on circPRKCI in human malignant tumors, including esophageal cancer, gastric cancer (GC), lung cancer, glioma, thyroid cancer, bladder cancer, hepatocellular carcinoma (HCC), laryngeal squamous cell carcinoma (LSCC), and cervical cancer (CC) (5–18). The circPRKCI oncogene can act as a sponge for many tumor suppressor microRNAs (miRNAs), such as miR-1324, miR-545, miR-589, miR-1304, miR-3680-3p, miR-1182, and miR-186-5p. Overexpression of circPRKCI can promote the progression of tumors by regulating mRNAs or certain signaling pathways, including the Wnt/β-catenin or phosphatidylinositol-3-kinase (PI3K)/AKT pathway. Liu et al. found through a meta-analysis that the high expression of circPRKCI leads to lower overall survival and poorer clinical features, indicating that circPRKCI may be a new target for prediction and prognostic evaluation in human cancer (19). The main purpose of this study is to comprehensively elucidate the specific biological mechanisms and functions of circPRKCI in various human tumors, and evaluate its biological significance.

### 1.2 Generation and subtype of circRNA

CircRNAs are derived from a non-canonical and alternative form of pre-mRNA back-splicing. Different from ordinary linear RNA, the 5′ and 3′ ends join together to form a continuous covalent closed loop, which increases both the stability of circRNA and its resistance to exonuclease-mediated degradation (20). CircRNAs can be divided into three main types: exons [exonic circRNA (EcircRNA)], introns [intronic circRNA (ciRNA)], and both [exon–intron circRNA (EIciRNA)] (21). In contrast to the splice junction of linear RNA, the looping mechanism of circRNAs includes exon lasso circularization and intron pairwise circularization (22).

### 1.3 Function of circRNAs

circRNAs influence RNA polymerase II (RNA Pol II) transcription by forming R-loops with their producing loci and co-activating transcription factors to modulate transcription (23). Unlike most RNAs involved in translation, circRNAs play a role in regulating gene expression. CircRNAs, together with other competing endogenous RNAs (ceRNAs), are considered sponges of miRNA. CircRNAs have more binding sites, which enable them to specifically bind to miRNAs (24). MiRNAs are small RNAs (length of approximately 20 nucleotides) that are mainly responsible for regulating the mRNA transcription of target genes (25). Most circRNAs contain conserved miRNA-binding sites that sponge miRNAs (26), which can specifically recognize and bind miRNA response elements to negatively regulate miRNA expression (27). A variety of circRNAs can interact with different miRNAs to regulate the occurrence and progression of tumors. CircRNAs can regulate the expression of parental genes by affecting their pre-mRNA. For example, Ashwal et al. identified conserved muscleblind (MBL) binding sites in circRNA MBL and its flanking introns, which are firmly and specifically bound by MBL (28). CircRNAs can bind to RNA binding proteins through their binding sites (29). Traditionally, circRNAs are considered endogenous non-coding RNA and cannot recruit ribosomes, but circRNAs containing an open reading frame driven by the internal ribosome entry site can directly bind to ribosomes and induce 5′-cap-independent translation (30, 31).

## 2 Clinical significance of circPRKCI in various tumors

CircRNAs have been demonstrated to act as molecular “sponges” to adsorb miRNAs and regulate the expression of downstream genes. This knowledge has prompted researchers to explore additional functional mechanisms of circRNAs in various diseases, especially in tumors (32). Further functional mechanisms of tumor-related circRNAs have been discovered, which can affect the progression of tumors by binding with proteins and even serving as a template for transcription, seemingly consistent with the traditional view (30, 31, 33, 34).

CircPRKCI deregulated by copy number variation is relatively novel and is the outcome of back-splicing of exons 15 and 16 of the PRKCI gene, located at the 3q26.2 amplicon (35). The abundance of circPRKCI in tumor tissues and adjacent non-tumor tissues was measured using quantitative real-time polymerase chain reaction (qRT-PCR), and the difference in the expression of circPRKCI between different TNM stages was detected using chromosome *in situ* hybridization. Fluorescence *in situ* hybridization (FISH) detected the subcellular localization of circPRKCI, specifically in the cytoplasm. However, Qi et al. detected abundant circPRKCI in the blood and observed no significant differences between patients with liver cancer and healthy individuals; this may reflect the fact that only a few studies have reported the expression of circPRKCI in plasma (12). Conforming to the subcellular localization and copy number of circPRKCI is beneficial for predicting cellular functions. The location can be detected using FISH and qRT-PCR after separating the nuclear and cytoplasmic components from the total RNA, and the copy number can be detected by CT values from qRT-PCR.

Many recent studies have confirmed the overexpression of circPRKCI in various malignant tumor tissues, including esophageal, liver, lung, cervical, thyroid, glioma, gastric, bladder, and laryngeal cancers. In one study, the expression of circPRKCI in primary lung adenocarcinoma tissues was 6.89 times higher than that in adjacent non-tumor tissues and was positively correlated with tumor size and TNM stage (7). The expression level of circPRKCI in esophageal squamous cell carcinoma (ESCC) tissue was 6.89 times higher than the expression in adjacent non-tumor tissues, and the upregulation of circPRKCI was significantly associated with poor tumor differentiation and a higher TNM stage (17). Another study demonstrated that circPRKCI was related to stronger lymph node metastasis and distant metastasis of laryngeal squamous cell cancer (LSCC) (10). Through the same methods, circPRKCI was observed to positively correlate with adverse clinical characteristics of other tumors, such as poor TNM stage, lymph node metastasis, and distant metastasis of HCC, cervical carcinoma, thyroid carcinoma, and glioma. In addition, in liver cancer, CC, GC, thyroid cancer, LSCC, lung cancer, and glioma, high expression levels of circPRKCI are closely related to a low overall survival rate of patients with tumors.

## 3 Roles of circPRKCI in various malignant tumors

**Figure 2:**
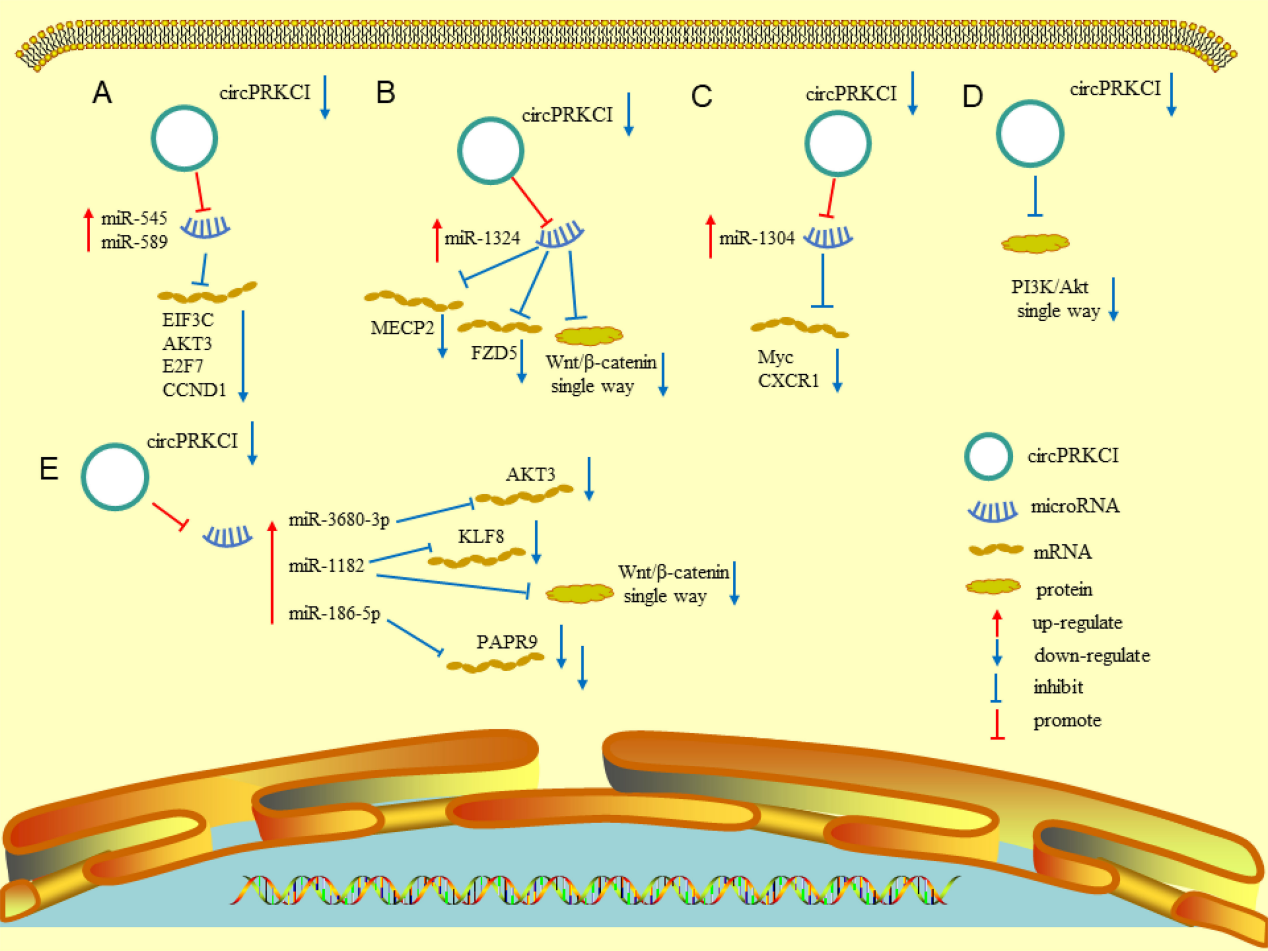
Various molecular mechanisms involved in circPRKCI. **(A)** Downregulation of circPRKCI can promote the expression of miR-545 and miR-589, and then inhibit the expression of EIF3C, AKT3, E2F7, and CCND1. **(B)** Downregulation of circPRKCI can promote the expression of miR-1324, and then inhibit the expression MECP2, FZD5, and Wnt/β-catenin pathway. **(C)** Downregulation of circPRKCI can promote the expression of mir-1304, and then inhibit the expression of myc and CXCR1. **(D)** Downregulation of circPRKCI could inhibit the expression of PI3K/AKT pathway. **(E)** Downregulation of circPRKCI can promote the expression of miR-3680-3p, miR-118, and miR-186-5p, and then inhibit the expression of AKT3, KLF8, PAPR9, and Wnt/β-catenin pathway.

Many recent studies have documented the involvement of circRNAs in tumorigenesis, progression, and metastasis through various mechanisms (36). The role of circPRKCI has been investigated in various cancers, including adenocarcinoma and squamous cell carcinoma. Understanding the functional mechanisms may benefit the diagnosis and treatment of related malignant tumors. The following sections detail the research on the mechanisms of circPRKCI in various tumors. **Figure 2** and **Tables 1**, **2** summarize the biomolecular mechanisms of circPRKCI in various malignant tumors.

**Table 1:** Clinical significance of high expression of circPRKCI in different tumors.

Tumor type	Samples normal:tumor	Clinicopathological features	Detection *p*-value	TNM *p*-value	LNM *p*-value	DM *p*-value	OS *p*-value	DFS *p*-value	Follow-up (months)	Ref.
NSCLC	(159:159)	High TNM, LNM, and DM Low OS	qRT-PCR *p* < 0.001	*p* = 0.003	*p* < 0.001	*p* = 0.017	*p* = 0.001	*-*	60	(5)
NSCLC	(79:79)	High TNM and positive LNM Low OS	qRT-PCR *p* < 0.05	*p* = 0.012	*p* = 0.018	–	*-*	*p* < 0.05	80	(6)
Lung cancer	(60:60)	High TNM stage Low OS	qRT-PCR *p* < 0.01	*p* < 0.01	*-*	*-*	*p* = 0.023	*-*	60	(8)
LSCC	(40:40)	Positive LNM and DM Low OS	qRT-PCR *p* < 0.001	*-*	*p* = 0.001	*p* = 0.004	*p* < 0.05	*-*	90	(10)
HCC	(49:49)	Low OS	qRT-PCR *p* < 0.001	*-*	*-*	*-*	*p* = 0.021	*-*	60	(11)
Gastric cancer	(50:50)	High TNM, Positive DM Low OS	qRT-PCR *p* < 0.01	*p* < 0.01	*-*	*p* < 0.05	*p* = 0.031	*-*	60	(14)
ESCC	(51:51)	High TNM	qRT-PCR *p* = 0.011	*p* = 0.021	*-*	*-*	*-*	*-*	*-*	(17)
Cervical carcinoma	(19:61)	Positive LNM Low OS	qRT-PCR *p* < 0.05	*-*	*p* < 0.05	*-*	*p* < 0.05	*-*	60	(37)
Bladder cancer	(54:54)	Positive LNM and DM Low 5-year OS and DFS	qRT-PCR *p* < 0.001	–	*p* = 0.004	*p* < 0.01-	*p* < 0.05	*p* < 0.05	60	(38)
Glioma	(157:157)	Low OS	qRT-PCR *p* < 0.01	*-*	*-*	*-*	*p* = 0.021	*p* = 0.011	60	(39)
Thyroid cancer	(57:57)	High TNM, Positive LNM Low OS	qRT-PCR *p* < 0.001	*p* = 0.031	*p* = 0.035	–	*p* < 0.05	–	80	(40)
Thyroid cancer	(50:50)	High TNM, Positive LNM Low OS	qRT-PCR *p* < 0.05	*p* < 0.05	*p* < 0.05	*-*	*p* = 0.0268	*-*	60	(41)

TNM, tumor-node-metastasis; LNM, lymph node metastasis; DM, distant metastasis; OS, overall survival; DFS, disease-free survival; NSCLC, non-small cell lung cancer; ESCC, esophageal squamous cell carcinoma; HCC, hepatocellular carcinoma; LSCC, laryngeal squamous cell cancer.

**Table 2:** Functions and mechanisms of circPRKCI in different tumors.

Tumor	Pattern of Expression	Accessed Cell	Regulators and Targets or Signaling Pathways	Biological Function	Ref.
Lung cancer	Up	A549, SCPA1 H1299	Sponge miR-545 and miR-589 and downregulate the expression of E2F7	↑Proliferation	(7)
Lung cancer	Up	A549 H1299	Bind miR-1324 and upregulate the expression of MECP2	↑Migration and invasion	(8)
NSCLC	Up	H358 H23	Act as ceRNA of miR-1182, then upregulate the expression KLF8 and activate the Wnt/β-catenin pathway	↑Invasion, migration, and proliferation	(9)
LSCC	Up	TU212 TU-686	Downregulate miR-1324	↑Proliferation and migration	(10)
HCC	Up	Hep3B HuH7	Downregulate miR-1324, then upregulate FZD5 and activate the Wnt/β-catenin pathway	↑Invasion, migration and proliferation	(11)
HCC	Up	HepG2	Act as ceRNA of miRNA-545, then downregulate AKT3 and regulate the RAC-γ serine/threonine-protein kinase pathway	↓Apoptosis ↑invasion	(12)
HCC	Up	HCCLM3	Downregulate miR-1294 and miR-186-5p, then upregulate the expression of FOXK1	↑Migration and invasion	(13)
Gastric cancer	Up	SGC-7901 MGC-803	Downregulate miR-545	↑Proliferation and invasion	(14)
ESCC	Up	TE-1 ECA-109	Sponge miR-3680-3p and then upregulate the expression of AKT3 protein	↑Proliferation and migration	(16)
ESCC	Up	ECA-109 KYSE450	Downregulate miR-186-5p and then upregulate the expression of PARP9	↑Cell activity and proliferation	(18)
Cervical carcinoma	Up	SiHa, CaSki, Hela, C4-1	Downregulate miR‐545 and then upregulate the expression of EIF3C	↑Invasion, migration, proliferation and EMT	(37)
Bladder cancer	Up	T24	Downregulate miR−1304 and then upregulate the expression of Myc	↑Proliferation, migration, and invasion	(38)
Breast cancer	Up	MDA-MB-231, BT-549, HCC-1937	Downregulate miR-545-3p and then upregulate WBP2, and activate the phosphorylation of AKT and the PI3K/AKT signaling pathway	↑Proliferation and migration	(42)
Glioma	Up	A172	Downregulate miR-545 and then upregulate the expression of E2F7 and RIG-1	↑Cell activity, migration, and proliferation	(43)
Glioblastoma	Up	U251, LN229, and A172	Act as an oncogenic promoter to activate the PI3K-AKT signaling pathway	↑Proliferation, EMT, migration, and apoptosis	(39)
Thyroid cancer	Up	K1 and SW579	Activate the PI3K/AKT pathway	↑Proliferation, EMT migration, and invasion	(40)
Thyroid cancer	Up	SW579 TPC-1	Downregulate miR−1304 and then upregulate the expression of CXCR1	↑Proliferation, migration, and invasion	(41)

### 3.1 Lung cancer

Qiu et al. reported that somatic copy number variations may promote cancer progression by regulating coding and non-coding transcripts. The authors identified a proto-oncogenic circRNA (circPRKCI) from the 3q26.2 amplicon through bioinformatics analysis. CircPRKCI was abnormally overexpressed in lung adenocarcinoma, partly because the copy number variations generated by amplification of 3q26.2 promoted the proliferation and tumorigenesis of lung adenocarcinoma. Through a series of *in vitro* and *in vivo* experiments, Qiu et al. reported that circPRKCI acts as a sponge for both miR-545 and miR-589, which can downregulate the expression of protumorigenic transcription factor E2F7 by binding to the 3′-UTR of E2F7 mRNA. CircPRKCI significantly promoted the proliferation of lung adenocarcinoma cells through the circPRKCI–miR-545/589–E2F7 axis (7). More importantly, the intratumoral injection of small interfering (si)-circPRKCI into a patient-derived tumor xenograft model from a female patient with lung adenocarcinoma significantly inhibited tumor growth *in vivo*. Furthermore, si-circPRKCI can enhance the inhibitory effect of epidermal growth factor receptor (EGFR)–tyrosine kinase inhibitors in HCC827 cells, which may enhance the therapeutic effect of these inhibitors in lung adenocarcinoma with EGFR mutations (7). Meng et al. reported that circPRKCI can bind to miRNA-1324, thereby increasing the expression of MECP2 and participating in the progression of lung cancer (8). Zhao et al. reported that circPRKCI acts as an oncogene and miRNA sponge in NSCLC. CircPRKCI can sponge miR-1182 and downregulate its expression of miR-1182, resulting in upregulation of KLF8 expression. Upregulation of miR-1182 can inactivate the Wnt/β-catenin pathway by repressing β-catenin, cyclin D1, and c-myc. However, overexpression of KLF8 can reverse the inhibition of miR-1182 in non-small cell lung cancer. Thus, circPRKCI can promote the progression and development of this cancer by the miR-1182/KLF8 axis and activation of the Wnt/β-catenin pathway (9). These studies confirm that circPRKCI is crucial for the occurrence of lung adenocarcinoma and may be a potential therapeutic target in patients with lung adenocarcinoma.

### 3.2 Laryngeal squamous cell carcinoma

The expression of circPRKCI is abnormally upregulated in LSCC and negatively correlates with the expression of miR-1324. Compared to adjacent non-tumor tissues, the expression level of miR-1324 in LSCC tissues is significantly reduced, as observed in LSCC cell lines. Subsequent mechanistic studies confirmed the specific binding sites of circPRKCI for miR-1324 and that circPRKC1 can sponge miR-1324. Knockdown of circPRKCI can inhibit the proliferation and migration of LSCC cell lines. The downregulation of miR-1324 can reverse the influence of circPRKCI knockdown. The authors described that circPRKCI acts as an oncogene in LSCC by downregulating the expression of miR-1324 (10).

### 3.3 Hepatocellular carcinoma

Qian et al. found that the expression of circPRKCI was upregulated in liver cancer. Knockdown of circPRKCI in liver cancer cells can inhibit proliferation, migration, and invasion of the cells, and induce apoptosis. In terms of the mechanism, the authors identified a putative binding site in circPRKCI for miR-1324, which could inhibit the expression of miR-1324. Further investigation of the downstream target of miR-1324 identified a putative binding site in the 3′-UTR of FZD5 for miR-1324, which was confirmed using luciferase activity reporter assays. This target promotes the progression of HCC by activating the Wnt/β-catenin signaling pathway. The effect of circPRKCI knockdown in HCC cells can be reversed by overexpressing FZD or downregulating miR-1324; both actions can enhance cell proliferation, migration, and invasion. As a result, circPRKCI can promote the progression of HCC through the circPRKCI/miR-1324/FZD5/Wnt/β-catenin signaling pathway axis, which may be a potential therapeutic target for HCC (11). Qi et al. reported that the expression of circPRKCI can influence the progression and invasiveness of HepG2 cells, which act as a sponge for miRNA-545. Overexpressing circPRKCI and miR-545 can decrease the expression of AKT3 protein. The findings indicate that circPRKCI participates in the pathogenesis of HCC through the RAC-γ serine/threonine-protein kinase pathway along with miR-545 (12). In another study, circPRKCI overexpression promoted the viability, invasion, and migration of HCC cells by sponging miR-1294 and miR-186-5p and upregulating FOXK1. Overexpression of FOXK1 can increase the levels of glucose and lactic acid, which can provide energy for the proliferation and metastasis of HCC cells. This effect can be reversed by circPRKCI knockdown. The findings indicate that circPRKCI regulates progression and glycolysis through the circPRKCI/miR-1294/miR-186-5p/FOXK1 axis (13).

### 3.4 Gastric cancer

Compared to adjacent normal tissues, the expression levels of circPRKCI are upregulated in gastric tissues and GC cell lines. A mechanistic study revealed that miR-545 has the potential to bind to circPRKCI, which was confirmed using a dual-luciferase reporter gene assay. The expression of miR-545 was decreased dramatically when circPRKCI was overexpressed. The authors described that the knockdown of circPRKCI can attenuate the proliferation and invasion of GC cells, but the downregulation of miR-545 can partially reverse this inhibitory effect. Another study revealed that circPRKCI can sponge miR-545 and regulate the progression of GC (14). Adopting the Gene Expression Omnibus database to detect the expression data and the RobustRankAggreg algorithm to integrate the differentially expressed circRNAs from the four datasets, He et al. reported that the upregulation of circPRKCI may correlate with GC, which may be related to the blockade of the Wnt and calcium-modulating pathways. Gene Ontology and Kyoto Encyclopedia of Genes and Genomes analyses revealed that circPRKCI participates in the genesis and progression of GC through the circPRKCI/miR-4705/BMPR1B axis (15).

### 3.5 Esophageal cancer

Shi et al. reported that the expression level of circPRKCI in ESCC was higher than that in the adjacent normal tissues. *In vitro* experiments verified that the high expression of circPRKCI promoted the proliferation and migration of ESCC cells. The authors further explored the role of circPRKCI in ESCC and identified a binding site between miR-3680-3p and circPRKCI that can sponge miR-3680-3p. The expression of AKT3 was upregulated, resulting in increased migration and proliferation of ESCC cells. The findings indicate that circPRKCI could promote the progression of ESCC via the circPRKCI/miR‐3680‐3p-AKT3 axis (16). Abnormal circPRKCI expression has been observed in esophageal cancer and ESCC cells. The knockdown of circPRKCI in loss-of-function experiments significantly decreased the cycle progression, viability, and colony formation abilities of ECSS cells. However, the upregulation of poly(ADP-ribose) polymerase 9 (PARP9) reversed the effect of circPRKCI knockdown in ESCC cells. The starBase3.0 database predicted that miR-186-5p is a potential target of circPRKCI, which was confirmed using a dual-luciferase reporter assay. CircPRKCI acts as a ceRNA for miR-186-5p and regulates the expression of PARP9 to regulate the progression of ESCC. Furthermore, the expression of circPRKCI negatively correlated with the sensitivity of ESCC cells to radiotherapy. Knockdown of circPRKCI can improve the radiosensitivity of ESCC cells, but the overexpression of PARP9 can offset this effect (18).

### 3.6 Cervical cancer

Hu et al. reported that the expression of circPRKCI was upregulated in CC and that the proliferation, migration, invasion, and epithelial–mesenchymal transition (EMT) levels of CC cells were downregulated after the downregulation of circPRKCI. *In vitro* and *in vivo* experiments have demonstrated the positive correlation of circPRKCI with the growth of CC tumors. Subsequent studies on the functional mechanisms of circPRKCI revealed that the expression level of miR-545 was upregulated following circPRKCI knockdown. MiR-545 could inhibit EIF3C by targeting its 3′-UTR region. Knockdown of circPRKCI reportedly suppressed the proliferation, migration, and invasion of CC cells. Functional recovery testing revealed that the overexpression of EIF3C could rescue this function. CircPRKCI participation in CC may be realized through the miR‐545/EIF3C axis. These findings may provide a new approach to the diagnosis and treatment of CC (37).

### 3.7 Bladder cancer

Liu et al. collected clinical samples of bladder cancer and determined that the expression level of circPRKCI was upregulated and that the high expression of circPRKCI was more likely to lead to a larger tumor diameter, higher tumor stage, and lymph node metastasis rate. After the downregulation of circPRKCI, a series of *in vitro* functional experiments confirmed that the proliferation, migration, and invasion of bladder cancer cells were significantly downregulated. The authors further analyzed the mechanism of circPRKCI in bladder cancer and reported that the knockdown of circPRKCI upregulated the expression of miR-1304. This relationship was confirmed using a dual-luciferase reporter assay. Myc was predicted to have a binding site for miR-1304, which was validated using a dual-luciferase reporter assay. The use of circPRKCI as a sponge for miR-1304 directly decreased the expression levels of miR-1304 in T24 cells and subsequently increased Myc expression. After the downregulation of circPRKCI, the expression of miR-1304 was upregulated, and the expression of Myc protein was further inhibited. The results implicate the circPRKCI/miR-1304/Myc axis as a new molecular target for the diagnosis and treatment of bladder cancer (38).

### 3.8 Breast cancer

Abnormal expression of circPRKCI was observed in breast cancer tissues and cell lines and was positively correlated with the proliferation and invasion ability of triple-negative breast cancer. Predictive circRNA databases were used to detect related miRNAs. The binding of miR-545-3p to circPRKCI is confirmed using RNA binding protein immunoprecipitation and luciferase reporter assays. Based on the potential binding sites between miR-545-3p and its target, dual-luciferase reporter assays were designed and observed that WBP2 was the target of miR-545-3p. Further mechanistic research demonstrated that the expression was positively correlated with the expression of circPRKCI and negatively correlated with that of miR-545-3p. As WBP2 can influence the phosphorylation of AKT, circPRKCI sponges miR-545-3p, upregulates the expression of WBP2, activates the PI3K/AKT signaling pathway, and participates in the proliferation and migration of triple-negative breast cancer cells (42). PI3K/AKT signaling is an important target for breast cancer treatment, and mutations in these pathways have been related to aggressive tumor behavior and treatment resistance (44). Prospective research has proven that the use of the PI3K inhibitor alpelisib can bring clinical benefits to patients with breast cancer, and has been approved for the targeted treatment of breast cancer (45). Therefore, circPRKCI is expected to become a potential therapeutic target for breast cancer.

### 3.9 Glioma

Zhang et al. reported that the expression in human glioma tissues and glioma cells was significantly increased compared to that in normal tissues. CircPRKCI knockdown inhibited the proliferation and migration of glioma cells and glioma growth in mice. However, circPRKCI overexpression reversed this effect. The authors described that the overexpression of miR-545, a known target of circPRKCI, could inhibit the expression of the transcription factors E2F7 and retinoic acid-inducible gene-I, and the progression of glioma cells. RIP assay results confirmed that miR-545 was the target of circPRKCI. Furthermore, the knockdown of circPRKCI could upregulate the expression of downstream target proteins. Downregulation of circPRKCI reduced the viability and inhibited the proliferation of glioma cells, which could be offset by miR-545 knockdown. In this study, circPRKCI acted as a ceRNA to inhibit the tumor-suppressive miR-545 and increased the expression of the downstream gene E2F7/RIG-1, which eventually promoted the progression of glioma (43).

Xin et al. reported that circPRKCI expression is upregulated in glioblastoma (GBM) tissues and cell lines, resulting in a lower overall survival rate in patients with glioma. Downregulation of circPRKCI inhibits the proliferation, migration, and EMT of GBM cells (39). Our mechanistic research demonstrated that the downregulation of circPRKCI can inhibit the expression of the PI3K/AKT pathway.

### 3.10 Thyroid carcinoma

Wang et al. reported that the expression of circPRKCI was upregulated in thyroid cancer through a series of *in vitro* experiments and that a high expression level of circPRKCI could lead to a poor prognosis in patients with thyroid cancer (40). Simultaneously, circPRKCI knockdown inhibited the proliferation, migration, invasion, and EMT of thyroid cancer cells. In terms of the mechanism, the expression level of the PI3K/AKT pathway is downregulated after the downregulation of circPRKCI. Liu et al. reported that circPRKCI can promote disease progression by forming ceRNA with miR-335 to increase the expression of E2F3 and that silencing of circPRKCI can suppress cell progression and glycolysis of papillary thyroid cancer (46). CircPRKCI is upregulated in thyroid cancer tissues and cell lines and promotes the progression of thyroid cancer. Knockdown of circPRKCI could upregulate the expression of miR-1304, which inhibited the proliferation and migration of thyroid cancer cells. A dual-luciferase reporter assay confirmed the potential binding sites between miR-1304 and CXCR1. Knockdown of circPRKCI could inhibit the expression of CXCR1, but this effect could be reversed by downregulation of miR-1304. Another study demonstrated that circPRKCI regulates the progression of thyroid cancer through the circPRKCI/miR-1304/CXCR1 axis (41).

## 4 Discussion

Although research on the role of circRNAs in human malignant tumors has progressed in recent years, many problems require further exploration. First, most publicly available tumor-related RNA-seq datasets use the poly A purification method to enrich mRNA, whereas circRNAs that lack poly A tails naturally may not have been studied. In addition, circRNAs can be used as oncogenes or tumor suppressors in various human cancers, and carcinogenic circRNAs play a carcinogenic role in a manner that does not interfere with linear mRNA expression (47). Moreover, circRNAs need to be included in individual treatment schemes in the future; therefore, it is necessary to further explore the precise mechanism of circRNAs in the occurrence and progression of cancer. Finally, a standard naming system is crucial for the standardized arrangement of circRNAs.

As an oncogene, circPRKCI plays an important role in the development of human malignant tumors. CircPRKCI has specific biological functions and participates in various cancers via several molecular mechanisms. High expression levels of circPRKCI in tumor tissues can lead to a larger tumor diameter, higher TNM stage, positive lymph node metastasis and distant metastasis, poor tumor classification, and lower overall survival rate of patients with tumors. CircPRKCI can act as a molecular sponge of miR-1324, miR-3680-3p, miR‐545, miR-1182, miR−1304, miR-186-5p, and miR-589 by specifically binding to these miRNAs. CircPRKCI competes with the downstream targets of these miRNAs, including MECP2, AKT3, EIF3C, FZD5, E2F7, KLF8, Myc, CXCR1, PARP9, and CCND1. These circRNA–miRNA–mRNA networks jointly regulate the progression of malignant tumors. In addition, circPRKCI can target the expression level of certain classical signaling pathways, including the Wnt/β-catenin and PI3K/AKT signaling pathways. CircPRKCI is involved in biological processes and molecular mechanisms regulating tumor proliferation, migration, invasion, and EMT, and may become a potential target for human cancer diagnosis and treatment in the future.

Although great progress has been made in research on circPRKCI in various human malignant tumors, its clinical application and mechanism of action still require further study. The difference in the abundance of circPRKCI is more pronounced in tissue samples, and no significant difference has been observed in the plasma of patients with tumors because few studies have focused on serum abundance. Qi et al. (12) detected the abundance of circPRKCI in plasma and observed no significant difference between patients with liver cancer and healthy individuals. Current research on circPRKCI has been limited to miRNA sponges. Therefore, future studies are needed to explore additional mechanisms, such as whether they can interact with related proteins or encode related proteins to promote the occurrence and development of various tumors.

This review has comprehensively discussed the biological function and molecular mechanism of circPRKCI in various malignant tumors. However, a drawback is that most studies analyzed the relationship between circPRKCI and the clinicopathological characteristics of patients with tumors through limited tumor tissues and normal control tissues. Additionally, the ethnic groups of these tissues were not diverse. Therefore, it is necessary to include more ethnic groups and larger sample sizes to further explore the characteristics of circPRKCI. However, the biological function and molecular mechanism of circPRKCI in other common malignant tumors, such as colorectal cancer, osteosarcoma, and endometrial cancer, remain unclear and require further exploration.

This paper reviews the latest research on circPRKCI, focusing on its clinical value and biological functions in various human malignant tumors. As an oncogene, circPRKCI plays an important role in specific mechanisms and biological functions via the circRNA–miRNA–mRNA network. In future studies, circPRKCI may serve as a novel tumor marker and provide a new target for the diagnosis and treatment of malignant tumors.

## Author contributions

SQ: Conceptualization, Data curation, Formal analysis, Investigation, Methodology, Project administration, Resources, Software, Supervision, Validation, Visualization, Writing – original draft, Writing – review & editing. KZ: Conceptualization, Data curation, Formal analysis, Methodology, Project administration, Supervision, Validation, Writing – review & editing. SC: Conceptualization, Investigation, Methodology, Software, Supervision, Writing – review & editing. SY: Methodology, Project administration, Software, Writing – review & editing.
